# 4-Amino­pyridinium hydrogen succinate

**DOI:** 10.1107/S1600536809006990

**Published:** 2009-03-14

**Authors:** Hoong-Kun Fun, Jain John, Samuel Robinson Jebas, T Balasubramanian

**Affiliations:** a X-ray Crystallography Unit, School of Physics, Universiti Sains Malaysia, 11800 USM, Penang, Malaysia; b Department of Physics, National Institute of Technology, Tiruchirappalli 620015, India

## Abstract

In the title salt, C_5_H_7_N_2_^+^·C_4_H_5_O_4_^−^, the asymmetric unit comprises an amino­pyridinium cation and a hydrogen succinate anion as protonation of the aromatic N atom of the 4-amino­pyridine mol­ecule has occurred. The crystal packing is stabilized by inter­molecular O—H⋯O and N—H⋯O hydrogen bonds that lead to a two-dimensional array. Short C—H⋯O contacts are also present.

## Related literature

For the biological activity of 4-amino­pyridine, see: Judge & Bever (2006 ▶); Schwid *et al.* (1997 ▶); Strupp *et al.* (2004 ▶). For the applications of succinic acid, see: Sauer *et al.* (2008 ▶); Song & Lee (2006 ▶); Zeikus *et al.* (1999 ▶). For related structures, see: Chao & Schempp (1977 ▶); Anderson *et al.* (2005 ▶); Bhattacharya *et al.* (1994 ▶); Karle *et al.* (2003 ▶); Gopalan *et al.* (2000 ▶); Leviel *et al.*, (1981 ▶). For stability of the temperature controller, see: Cosier & Glazer (1986 ▶).
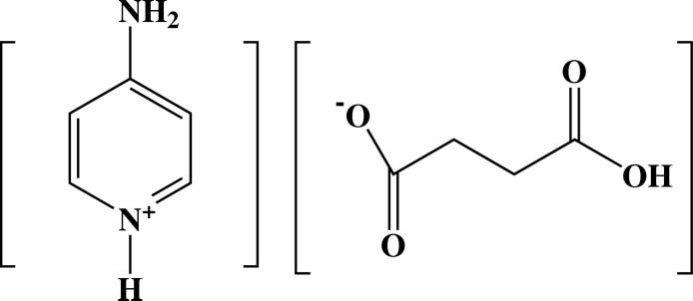


## Experimental

### 

#### Crystal data

C_5_H_7_N_2_^+^·C_4_H_5_O_4_^−^
*M* *_r_* = 212.21
Monoclinic, 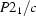

*a* = 6.5443 (3) Å
*b* = 22.2867 (11) Å
*c* = 7.1112 (4) Å
β = 114.587 (4)°
*V* = 943.13 (8) Å^3^
*Z* = 4
Mo *K*α radiation
μ = 0.12 mm^−1^
*T* = 100 K
0.38 × 0.14 × 0.08 mm

#### Data collection

Bruker SMART APEXII CCD area-detector diffractometer
Absorption correction: multi-scan (*SADABS*; Bruker, 2005 ▶) *T*_min_ = 0.956, *T*_max_ = 0.991
7174 measured reflections
2176 independent reflections
1483 reflections with *I* > 2σ(*I*)
*R*_int_ = 0.066

#### Refinement

*R*[*F* ^2^ > 2σ(*F* ^2^)] = 0.062
*wR*(*F* ^2^) = 0.150
*S* = 1.06
2176 reflections
148 parameters
H atoms treated by a mixture of independent and constrained refinement
Δρ_max_ = 0.44 e Å^−3^
Δρ_min_ = −0.46 e Å^−3^

### 

Data collection: *APEX2* (Bruker, 2005 ▶); cell refinement: *SAINT* (Bruker, 2005 ▶); data reduction: *SAINT*; program(s) used to solve structure: *SHELXTL* (Sheldrick, 2008 ▶); program(s) used to refine structure: *SHELXTL*; molecular graphics: *SHELXTL*; software used to prepare material for publication: *SHELXTL* and *PLATON* (Spek, 2009 ▶).

## Supplementary Material

Crystal structure: contains datablocks global, I. DOI: 10.1107/S1600536809006990/tk2378sup1.cif

Structure factors: contains datablocks I. DOI: 10.1107/S1600536809006990/tk2378Isup2.hkl

Additional supplementary materials:  crystallographic information; 3D view; checkCIF report

## Figures and Tables

**Table 1:** Hydrogen-bond geometry (Å, °)

*D*—H⋯*A*	*D*—H	H⋯*A*	*D*⋯*A*	*D*—H⋯*A*
O3—H1*O*3⋯O2^i^	1.09	1.40	2.482 (2)	176
N1—H1*N*1⋯O3^ii^	0.94 (3)	2.00 (3)	2.926 (3)	168 (3)
N2—H1*N*2⋯O1^iii^	0.90 (3)	2.59 (3)	3.115 (3)	118 (3)
N2—H1*N*2⋯O2^iii^	0.90 (3)	1.92 (3)	2.810 (3)	174 (3)
N1—H2*N*1⋯O4	0.85 (3)	2.08 (3)	2.934 (3)	175 (2)
C1—H1*A*⋯O4^ii^	0.93	2.54	3.440 (3)	164
C2—H2*A*⋯O1^iii^	0.93	2.39	3.041 (3)	127
C3—H3*A*⋯O1^iv^	0.93	2.31	3.222 (3)	166

Symmetry codes: (i) 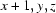; (ii) 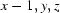; (iii) 
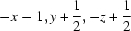
; (iv) 
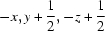
.

**Fig. 1.**
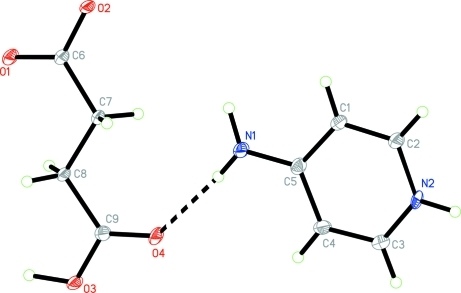
The molecular structure of (I), showing 50% probability displacement ellipsoids and the atom numbering scheme. The dashed line indicates hydrogen bonding.

**Fig. 2.**
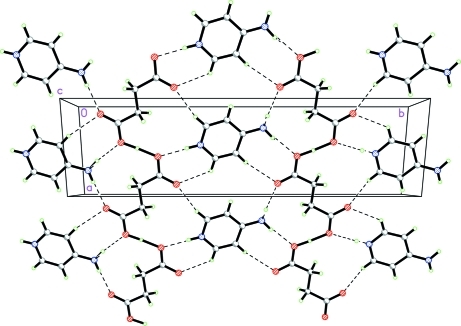
A 2-D supramolecular layer in (I), viewed along the c axis. Dashed lines indicate the hydrogen bonding.

## supplementary crystallographic information

### Comment

4-Aminopyridine (Fampridine) is used clinically in Lambert-Eaton myasthenic syndrome and multiple sclerosis because by blocking potassium channels it prolongs action potentials thereby increasing transmitter release at the neuromuscular junction (Judge & Bever, (2006); Schwid *et al.*, 1997; Strupp *et al.*, 2004). The structure of 4-aminopyridine has been reported (Chao & Schempp, 1977) as has a redetermination (Anderson *et al.*, 2005). Succinic acid is a dicarboxylic acid and is a precursor for many chemicals of industrial importance (Zeikus *et al.*, 1999; Song & Lee, 2006). Succinic acid derivatives are mostly being used in chemicals, food and pharmaceuticals (Sauer *et al.*, 2008). The crystal structure of succinic acid has also been reported (Gopalan *et al.*, 2000; Leviel *et al.*, 1981). As an extension of our systematic study of hydrogen bonding patterns of 4-aminopyridine with carboxylic acids, the title compound (I) has been synthesized and the crystal structure determined.

The asymmetric unit of (I) (Fig. 1) contains a 4-aminopyridinium cation and a succinic acetate anion, indicating that proton transfer occurred during the co-crystallisation experiment. Protonation leads to the widening of C2–N2–C3 angle in the pyridine ring to 120.7 (2)°, compared to 115.25 (13)° in 4-aminopyridine (Anderson *et al.*, 2005). This type of protonation has been observed in various 4-aminopyridine acid complexes (Bhattacharya *et al.*, 1994; Karle *et al.*, 2003). Otherwise, the bond lengths and bond angles in 4-aminopyridinium cation are comparable to the values reported earlier for 4-aminopyridine (Chao & Schempp, 1977; Anderson *et al.*, 2005). The 4-aminopyridine ring is essentially planar with the maximum deviation from planarity being -0.011 (3) Å for atom C5. The bond lengths and bond angles of the succinic acetate are found to have normal values (Gopalan *et al.*, 2000; Leviel *et al.*, 1981).

The crystal packing is consolidated by O—H···O and N—H···O intermolecular hydrogen bonds (Table 1) supported by C—H···O contacts. An intramolecular N—H···O hydrogen bond stabilises the conformation of the molecule. The molecules aggregate to form a 2-D array parallel to the *ab*-plane (Fig. 2).

### Experimental

Equimolar quantities of 4-aminopyridine (0.094 g, 1 mmol) and succinic acid (0.118 g, 1 mmol) were dissolved in  ethanol (10 ml) and water (10 ml), respectively. The aqueous solution of succinic acid was added drop wise to the solution of 4-aminopyridine and stirred well for 4 h. The solution is refluxed at 343°K for 6 h. Colourless crystals were harvested after one month of solvent evaporation.

### Refinement

The N-bound H atoms were located from the Fourier map and are allowed to refine freely (N-H = 0.85 - 0.94 (3) Å). The O-bound H atom was located from the Fourier map and fixed in that position, with O—H = 1.09 Å, and allowed to refine with *U*_iso_(H) = 1.2*U*_eq_(O). All other H atoms were placed in calculated positions, with C—H = 0.93 — 0.97 Å, and refined using a riding model with *U*_iso_(H) = 1.2*U*_eq_(C).

### Crystal data

**Table:**

C_5_H_7_N_2_^+^·C_4_H_5_O_4_^−^	*F*(000) = 448
*M**_r_* = 212.21	*D*_x_ = 1.494 Mg m^−^^3^
Monoclinic, *P*2_1_/*c*	Mo *K*α radiation, λ = 0.71073 Å
Hall symbol: -P 2ybc	Cell parameters from 1804 reflections
*a* = 6.5443 (3) Å	θ = 3.4–30.1°
*b* = 22.2867 (11) Å	µ = 0.12 mm^−^^1^
*c* = 7.1112 (4) Å	*T* = 100 K
β = 114.587 (4)°	Plate, colourless
*V* = 943.13 (8) Å^3^	0.38 × 0.14 × 0.08 mm
*Z* = 4	

### Data collection

**Table:**

Bruker SMART APEXII CCD area-detector diffractometer	2176 independent reflections
Radiation source: fine-focus sealed tube	1483 reflections with *I* > 2σ(*I*)
graphite	*R*_int_ = 0.066
φ and ω scans	θ_max_ = 27.5°, θ_min_ = 3.3°
Absorption correction: multi-scan (*SADABS*; Bruker, 2005)	*h* = −8→8
*T*_min_ = 0.956, *T*_max_ = 0.991	*k* = −28→28
7174 measured reflections	*l* = −9→8

### Refinement

**Table:**

Refinement on *F*^2^	Primary atom site location: structure-invariant direct methods
Least-squares matrix: full	Secondary atom site location: difference Fourier map
*R*[*F*^2^ > 2σ(*F*^2^)] = 0.062	Hydrogen site location: inferred from neighbouring sites
*wR*(*F*^2^) = 0.150	H atoms treated by a mixture of independent and constrained refinement
*S* = 1.06	*w* = 1/[σ^2^(*F*_o_^2^) + (0.0786*P*)^2^] where *P* = (*F*_o_^2^ + 2*F*_c_^2^)/3
2176 reflections	(Δ/σ)_max_ = 0.001
148 parameters	Δρ_max_ = 0.44 e Å^−^^3^
0 restraints	Δρ_min_ = −0.46 e Å^−^^3^

### Special details

**Table:**

Experimental. The crystal was placed in the cold stream of an Oxford Cyrosystems Cobra open-flow nitrogen cryostat (Cosier & Glazer, 1986) operating at 100.0 (1) K.
Geometry. All e.s.d.'s (except the e.s.d. in the dihedral angle between two l.s. planes) are estimated using the full covariance matrix. The cell e.s.d.'s are taken into account individually in the estimation of e.s.d.'s in distances, angles and torsion angles; correlations between e.s.d.'s in cell parameters are only used when they are defined by crystal symmetry. An approximate (isotropic) treatment of cell e.s.d.'s is used for estimating e.s.d.'s involving l.s. planes.
Refinement. Refinement of *F*^2^ against ALL reflections. The weighted *R*-factor *wR* and goodness of fit *S* are based on *F*^2^, conventional *R*-factors *R* are based on *F*, with *F* set to zero for negative *F*^2^. The threshold expression of *F*^2^ > σ(*F*^2^) is used only for calculating *R*-factors(gt) *etc*. and is not relevant to the choice of reflections for refinement. *R*-factors based on *F*^2^ are statistically about twice as large as those based on *F*, and *R*- factors based on ALL data will be even larger.

### Fractional atomic coordinates and isotropic or equivalent isotropic displacement parameters (Å^2^)

**Table:**

	*x*	*y*	*z*	*U*_iso_*/*U*_eq_	
O1	−0.1281 (3)	0.19522 (7)	0.2994 (3)	0.0174 (4)	
O2	−0.4204 (3)	0.24560 (7)	0.2995 (3)	0.0153 (4)	
O3	0.4285 (3)	0.34855 (7)	0.2828 (3)	0.0176 (4)	
O4	0.1374 (3)	0.40250 (7)	0.2633 (3)	0.0189 (4)	
N1	−0.2764 (4)	0.44695 (9)	0.2808 (3)	0.0171 (5)	
N2	−0.4551 (3)	0.62442 (9)	0.2152 (3)	0.0151 (5)	
C1	−0.5396 (4)	0.52400 (10)	0.2605 (4)	0.0151 (5)	
H1A	−0.6369	0.4962	0.2769	0.018*	
C2	−0.5929 (4)	0.58295 (10)	0.2401 (4)	0.0155 (5)	
H2A	−0.7272	0.5953	0.2432	0.019*	
C3	−0.2614 (4)	0.60783 (10)	0.2064 (4)	0.0156 (5)	
H3A	−0.1701	0.6368	0.1864	0.019*	
C4	−0.1974 (4)	0.54940 (10)	0.2261 (4)	0.0159 (5)	
H4A	−0.0632	0.5387	0.2195	0.019*	
C5	−0.3347 (4)	0.50458 (10)	0.2570 (4)	0.0132 (5)	
C6	−0.2223 (4)	0.24291 (10)	0.3003 (4)	0.0124 (5)	
C7	−0.1087 (4)	0.30270 (9)	0.3032 (4)	0.0119 (5)	
H7A	−0.2001	0.3251	0.1798	0.014*	
H7B	−0.1002	0.3257	0.4220	0.014*	
C8	0.1262 (4)	0.29562 (10)	0.3127 (4)	0.0124 (5)	
H8A	0.2230	0.2786	0.4458	0.015*	
H8B	0.1206	0.2674	0.2067	0.015*	
C9	0.2288 (4)	0.35388 (10)	0.2832 (4)	0.0136 (5)	
H1O3	0.4901	0.3032	0.2838	0.016*	
H1N1	−0.378 (4)	0.4190 (13)	0.292 (4)	0.020 (7)*	
H1N2	−0.490 (5)	0.6636 (14)	0.204 (4)	0.027 (8)*	
H2N1	−0.155 (5)	0.4361 (11)	0.273 (4)	0.013 (6)*	

### Atomic displacement parameters (Å^2^)

**Table:**

	*U*^11^	*U*^22^	*U*^33^	*U*^12^	*U*^13^	*U*^23^
O1	0.0163 (9)	0.0058 (8)	0.0317 (11)	−0.0004 (6)	0.0114 (8)	−0.0008 (7)
O2	0.0120 (8)	0.0061 (8)	0.0294 (11)	−0.0011 (6)	0.0102 (8)	0.0000 (6)
O3	0.0139 (9)	0.0067 (8)	0.0366 (12)	0.0003 (6)	0.0149 (8)	0.0014 (7)
O4	0.0182 (9)	0.0057 (8)	0.0358 (12)	0.0021 (6)	0.0141 (8)	0.0021 (7)
N1	0.0141 (10)	0.0079 (10)	0.0326 (14)	0.0014 (8)	0.0131 (10)	0.0008 (8)
N2	0.0170 (11)	0.0035 (10)	0.0235 (13)	0.0024 (7)	0.0071 (9)	0.0001 (8)
C1	0.0144 (12)	0.0104 (12)	0.0225 (15)	−0.0008 (8)	0.0096 (11)	0.0020 (9)
C2	0.0142 (12)	0.0092 (12)	0.0240 (15)	0.0000 (8)	0.0089 (11)	0.0002 (9)
C3	0.0139 (12)	0.0131 (12)	0.0204 (14)	−0.0026 (9)	0.0076 (11)	0.0010 (10)
C4	0.0125 (12)	0.0111 (12)	0.0255 (15)	−0.0021 (8)	0.0093 (11)	−0.0014 (9)
C5	0.0147 (11)	0.0092 (11)	0.0137 (13)	−0.0011 (8)	0.0038 (10)	−0.0012 (9)
C6	0.0128 (11)	0.0082 (11)	0.0162 (14)	−0.0009 (8)	0.0060 (10)	0.0000 (9)
C7	0.0117 (11)	0.0067 (11)	0.0175 (14)	0.0002 (8)	0.0063 (10)	0.0005 (9)
C8	0.0115 (11)	0.0063 (11)	0.0205 (14)	−0.0005 (8)	0.0076 (10)	0.0004 (9)
C9	0.0122 (11)	0.0107 (12)	0.0195 (14)	−0.0002 (8)	0.0081 (11)	−0.0001 (9)

### Geometric parameters (Å, °)

**Table:**

O1—C6	1.230 (3)	C1—H1A	0.9300
O2—C6	1.295 (3)	C2—H2A	0.9300
O3—C9	1.313 (3)	C3—C4	1.357 (3)
O3—H1O3	1.0871	C3—H3A	0.9300
O4—C9	1.217 (3)	C4—C5	1.420 (3)
N1—C5	1.331 (3)	C4—H4A	0.9300
N1—H1N1	0.94 (3)	C6—C7	1.522 (3)
N1—H2N1	0.86 (3)	C7—C8	1.519 (3)
N2—C3	1.347 (3)	C7—H7A	0.9700
N2—C2	1.354 (3)	C7—H7B	0.9700
N2—H1N2	0.90 (3)	C8—C9	1.516 (3)
C1—C2	1.352 (3)	C8—H8A	0.9700
C1—C5	1.419 (3)	C8—H8B	0.9700
			
C9—O3—H1O3	116.8	N1—C5—C4	122.1 (2)
C5—N1—H1N1	118.3 (16)	C1—C5—C4	116.8 (2)
C5—N1—H2N1	119.4 (17)	O1—C6—O2	122.88 (19)
H1N1—N1—H2N1	122 (2)	O1—C6—C7	120.88 (19)
C3—N2—C2	120.7 (2)	O2—C6—C7	116.24 (18)
C3—N2—H1N2	118.4 (17)	C8—C7—C6	112.93 (18)
C2—N2—H1N2	120.9 (17)	C8—C7—H7A	109.0
C2—C1—C5	119.9 (2)	C6—C7—H7A	109.0
C2—C1—H1A	120.1	C8—C7—H7B	109.0
C5—C1—H1A	120.1	C6—C7—H7B	109.0
C1—C2—N2	121.4 (2)	H7A—C7—H7B	107.8
C1—C2—H2A	119.3	C9—C8—C7	113.79 (18)
N2—C2—H2A	119.3	C9—C8—H8A	108.8
N2—C3—C4	121.0 (2)	C7—C8—H8A	108.8
N2—C3—H3A	119.5	C9—C8—H8B	108.8
C4—C3—H3A	119.5	C7—C8—H8B	108.8
C3—C4—C5	120.2 (2)	H8A—C8—H8B	107.7
C3—C4—H4A	119.9	O4—C9—O3	121.5 (2)
C5—C4—H4A	119.9	O4—C9—C8	123.62 (19)
N1—C5—C1	121.0 (2)	O3—C9—C8	114.91 (18)
			
C5—C1—C2—N2	0.3 (4)	C3—C4—C5—C1	1.5 (4)
C3—N2—C2—C1	1.2 (4)	O1—C6—C7—C8	−2.1 (3)
C2—N2—C3—C4	−1.3 (4)	O2—C6—C7—C8	177.7 (2)
N2—C3—C4—C5	−0.1 (4)	C6—C7—C8—C9	171.3 (2)
C2—C1—C5—N1	178.7 (2)	C7—C8—C9—O4	3.3 (3)
C2—C1—C5—C4	−1.6 (4)	C7—C8—C9—O3	−177.5 (2)
C3—C4—C5—N1	−178.8 (2)		

### Hydrogen-bond geometry (Å, °)

**Table:**

*D*—H···*A*	*D*—H	H···*A*	*D*···*A*	*D*—H···*A*
O3—H1O3···O2^i^	1.09	1.40	2.482 (2)	176
N1—H1N1···O3^ii^	0.94 (3)	2.00 (3)	2.926 (3)	168 (3)
N2—H1N2···O1^iii^	0.90 (3)	2.59 (3)	3.115 (3)	118 (3)
N2—H1N2···O2^iii^	0.90 (3)	1.92 (3)	2.810 (3)	174 (3)
N1—H2N1···O4	0.85 (3)	2.08 (3)	2.934 (3)	175 (2)
C1—H1A···O4^ii^	0.93	2.54	3.440 (3)	164
C2—H2A···O1^iii^	0.93	2.39	3.041 (3)	127
C3—H3A···O1^iv^	0.93	2.31	3.222 (3)	166

Symmetry codes: (i) *x*+1, *y*, *z*; (ii) *x*−1, *y*, *z*; (iii) −*x*−1, *y*+1/2, −*z*+1/2; (iv) −*x*, *y*+1/2, −*z*+1/2.

## References

[bb1] Anderson, F. P., Gallagher, J. F., Kenny, P. T. M. & Lough, A. J. (2005). *Acta Cryst.* E**61**, o1350–o1353.

[bb2] Bhattacharya, S., Dastidar, P. & Guru Row, T. N. (1994). *Chem. Mater.* **6**, 531–537.

[bb3] Bruker (2005). *APEX2*, *SAINT* and *SADABS* (Version 2004/1). Bruker AXS Inc., Madison, Wisconsin, USA.

[bb4] Chao, M. & Schempp, E. (1977). *Acta Cryst.* B**33**, 1557–1564.

[bb5] Cosier, J. & Glazer, A. M. (1986). *J. Appl. Cryst.* **19**, 105–107.

[bb6] Gopalan, R. S., Kumaradhas, P., Kulkarani, G. U. & Rao, C. N. R. (2000). *J. Mol. Struct.* **521**, 97–106.

[bb7] Judge, S. & Bever, C. (2006). *Pharmacol. Ther.* **111**, 224–259. 10.1016/j.pharmthera.2005.10.006 16472864

[bb8] Karle, I., Gilardi, R. D., Chandrashekhar Rao, Ch., Muraleedharan, K. M. & Ranganathan, S. (2003). *J. Chem. Crystallogr.* **33**, 727–749.

[bb9] Leviel, J.-L., Auvert, G. & Savariault, J.-M. (1981). *Acta Cryst.* B**37**, 2185–2189.

[bb10] Sauer, M., Porro, D., Mattanovich, D. & Branduaradi, P. (2008). *Trends Biotechnol.* **26**, 100–108. 10.1016/j.tibtech.2007.11.006 18191255

[bb11] Schwid, S. B., Petrie, M. D., McDermott, M. P., Tierney, D. S., Mason, D. H. & Goodman, A. D. (1997). *Neurology*, **48**, 817–821. 10.1212/wnl.48.4.817 9109861

[bb12] Sheldrick, G. M. (2008). *Acta Cryst.* A**64**, 112–122. 10.1107/S0108767307043930 18156677

[bb13] Song, H. & Lee, S. Y. (2006). *Enzyme Microb. Technol.* **39**, 352–361.

[bb14] Spek, A. L. (2009). *Acta Cryst.* D**65**, 148–155. 10.1107/S090744490804362X PMC2631630 19171970

[bb15] Strupp, M., Kalla, R., Dichgans, M., Fraitinger, T., Glasauer, S. & Brandt, T. (2004). *Neurology*, **62**, 1623–1625. 10.1212/01.wnl.0000125691.74109.53 15136697

[bb16] Zeikus, J. G., Jain, M. K. & Elankovan, P. (1999). *Appl. Microbiol. Biotechnol.* **51**, 545–552.

